# Modified mix design development specification batched by volume from specified mix design by weight towards improved concrete production

**DOI:** 10.1016/j.mex.2020.100817

**Published:** 2020-02-20

**Authors:** Opeyemi Joshua, Kolapo O. Olusola, David O. Nduka, Anthony N. Ede, Oluwarotimi M. Olofinnade, Olorunmeye F. Job

**Affiliations:** a Department of Building Technology, Covenant University, Ota, Nigeria; b Department of Building, Obafemi Awolowo University, Ile-Ife, Nigeria; c Department of Civil Engineering, Covenant University, Ota, Nigeria; d Department of Building, University of Jos, Jos, Nigeria

**Keywords:** Portland cement concrete, Weight batching, Volume batching, Concrete mix-design, Standardized prescribed concrete

## Abstract

• Concrete production process is usually designed to meet certain fresh properties, target strength and durability requirement. This process is referred to as the mix design, which guides the quantity and proportions of the various constituent materials to produce the concrete. Concrete mix designs are usually done in accordance to specified standard procedures in codes developed by recognized institutions like the Building Research Establishments (BRE) [1]. Other acceptable mix design methods includes the three (3) equation and double coating methods in [[2], [3]]. Standardized prescribed mix designs are generally accepted designs that meet strength requirements in normal strength concrete class as specified in [[4], [5]]. Standardized Prescribed Concrete mixes have been designed and the characteristic strengths specified in the British standards [[4], [5]] and the specified concrete mix design is recommended to be batched by weight.
• Predominantly, mix designs are batched by volume within the study area and most developing countries which results to the production of less durable concrete than when batched by weight. This practice is due to the higher cost of acquiring the batching plants [6] employed in concrete production by medium to small scale construction firms.
• This study developed a method of deriving a mix design to be batched by volume from the specified mix designed by weight using a design chat developed from [[4], [5]]. Concrete can then be produced with the derived mixed design and batched by volume as though it was batched by weight from the specified mix design. This method eliminates the strength disparity by both batching methods and production of more durable concrete in most developing countries.

## Graphical abstract

**Figure:**
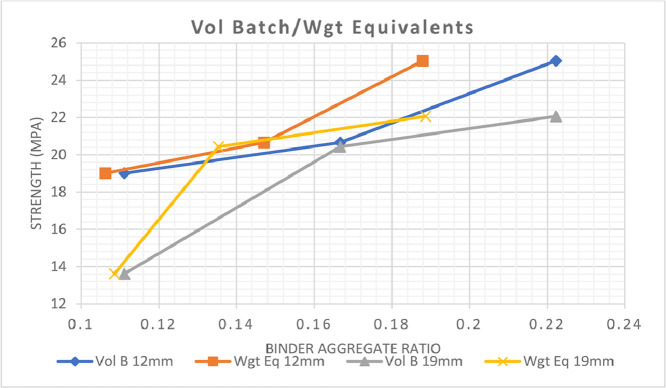

**Table:** Specification Table

Subject Area:	Engineering
More specific subject area:	Reinforced Concrete, Portland Cement, Concrete Strength, Mix-Design and Civil Engineering.
Method name:	Derivation of concrete mix design for volumetric batching from specified mix designed by weight from charts.
Name and reference of original method:	Mix design specification in [[4], [5]],
Resource availability:	Chart innovatively derived from mix designs and strength range specified in [[4], [5]]

## Method details [1–6]

Where:

Wgt B - Weight Batch
Vol B - Volume Batch
Vol B 12mm - Volume batched with 12 mm aggregate size
Wgt Eq 12mm - Weight equivalent of volume batched with 12 mm aggregate size
Vol B 19mm - Volume batched with 19 mm aggregate size
Wgt Eq 19mm - Weight equivalent of volume batched with 19 mm aggregate size
Wgt B 12mm - Weight batched with 12 mm aggregate size
Vol Eq 12mm - Volume equivalent of weight batched with 12 mm aggregate size
Wgt B 19mm - Weight batched with 19 mm aggregate size
Vol Eq 19mm - Volume equivalent of weight batched with 19 mm aggregate size

As observed in Table 1, the weight-equivalents of all binder-aggregate ratio batched by volume are all less than the actual weight-batched binder-aggregate ratio. This finding implies that all batches by volume contain more aggregate content by weight than when they are all batched originally by weight, hence, weaker concrete is expected in the batches by volume compared to when the same mix design is batched by weight. For instance, in Table 1, when fresh concrete with standardized prescribed mix-design of 1:2:4 with 12 mm aggregate size was batched by volume, the aggregate-binder ratio is 0.167 by volume batch. Comparatively, when the weight-equivalent mix-design of the same volume batch is determined, the same 1:2:4 batched by volume becomes 1:2.62:4.18 and 0.147 (binder-aggregate ratio) when viewed as a weight batch, and the concrete strength was 20.65 MPa, see Fig. 2. This implies that when the two mix-designs (same mix-design batched by volume and by weight) are compared from the weight perspective, the weight equivalent of the volume batch becomes 1:2.62:4.18 with binder aggregate ratio of 0.147 as against when the mix-design was batched by weight, 1:2:4 with binder aggregate ratio of 0.167 with a concrete strength of 22.47Mpa, see Fig. 3. This indicates that the volume-batched design mix in weight will contain more aggregate content than when it is originally batched by weight and this is evident in the strength variation. The more aggregate content in the volume batch will then imply a weaker concrete mix by virtue of the reduced binder content.

**Table 1:** Mix-design batch and their equivalent mix-designs in other batching method.

Aggt Size	Batch Type	Mix-design/Binder Aggt ratio	Coarse/Fine aggt. ratio	Mix ratio/Binder Aggt ratio	Coarse/Fine aggt. ratio	Mix ratio/Binder Aggt ratio	Coarse/Fine aggt. ratio
12mm	Volume	1:1.5:3 0.222	2.0	1:2:4 0.167	2.0	1:3:6 0.111	2.0
	Weight Equivalent	1:1.94:3.38 0.188	1.742	1:2.62:4.18 0.147	1.595	1:3.05:6.36 0.106	2.085
19m	Volume	1:1.5:3 0.222	2.0	1:2:4 0.167	2.0	1:3:6 0.111	2.0
	Weight Equivalent	1:1.99:3.31 0.189	1.663	1:2.83:4.56 0.135	1.611	1:2.87:6.34 0.109	2.209
12mm	Weight	1:1.5:3 0.222	2.0	1:2:4 0.167	2.0	1:3:6 0.111	2.0
	Volume Equivalent	1:1.48:2.6 0.245	1.757	1:1.6:2.88 0.223	1.8	1:2.25:4.07 0.158	1.808
19mm	Weight	1:1.5:3 0.222	2.0	1:2:4 0.167	2.0	1:3:6 0.111	2.0
	Volume Equivalent	1:1.6:2.8 0.227	1.75	1:1.21:2.30 0.172	1.9	1:2.71:4.47 0.139	1.649

The strength is related to the Standardized Prescribed Mix (SPC) mix-design by the binder aggregate ratio. According to [7], the more the binder content in a concrete mix, the better the strength property of the hardened concrete. The concrete cube strength will be discussed in relation to the binder aggregate ratio.

**Fig. 1:**
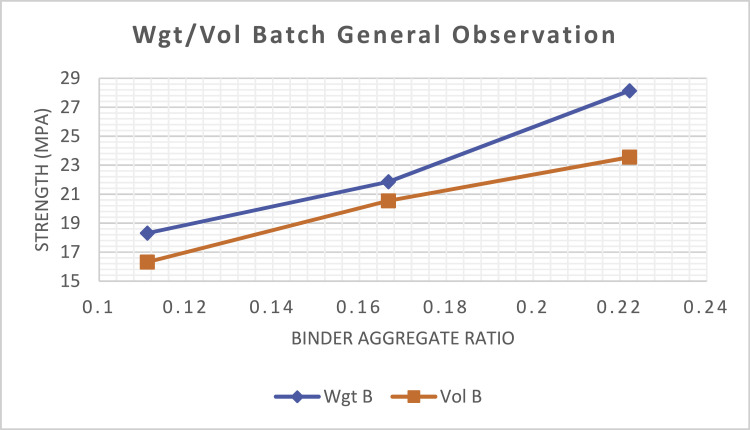
General compressive strengths of all weight and volume batches.

**Fig. 2:**
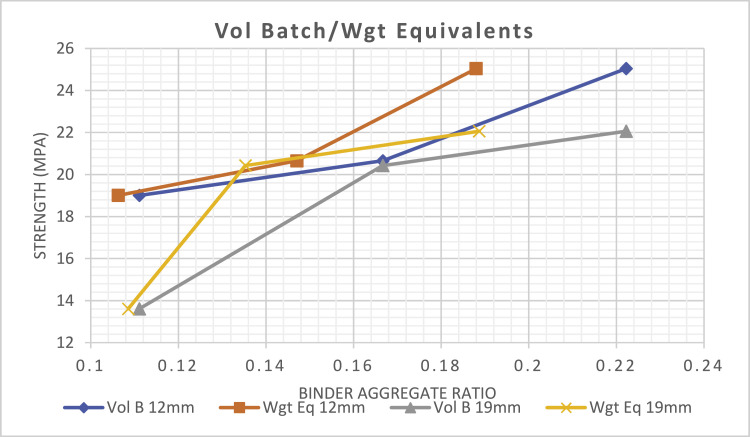
Shows volume batching and its weight equivalent.

**Fig. 3:**
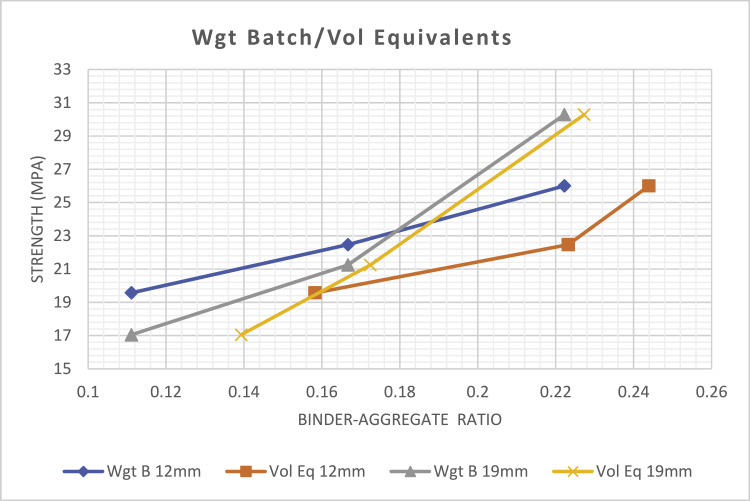
Shows weight batch and its volume equivalent.

Fig. 1 shows the result of the average compressive strength of all the concrete batched by volume and by weight. It shows that the strength of all concrete batched by weight produced superior strength to those batched by volume and this is confirmed in [[8], [9]]. Figs. 2 and 3 shows a relationship that exist between the strength of concrete batched by volume and by weight. This can be used as a rough guide to relate a weight batch to a volume batch mix-designs that will be equivalent to the design mix batched by weight. From Table 1, the average coarse to fine aggregate ratio is 1.776 when batched by weight and the volume-equivalent of the weight batch determined.

For instance, to prepare a mix-design to produce a concrete strength of 23 MPa with a 19 mm aggregate size. Since the goal is to obtain a mix-design to be batched by volume in a way as though it was designed to be batched by weight. The first step would be to identify the aggregate size to be used. Secondly, Fig. 3 would be used to determine a point on the Volume-quivalent curve of the weight-batch that corresponds with the target strength of 23 MPa. That point is 0.184 and this implies that the binder aggregate ratio is 0.184. The inverse of 0.184 would be 5.435 which would be equivalent to the sum of the fine and coarse aggregate ratios. Since the coarse to fine aggregate ratio would be 1.776, then the mix-design by volume batch that would produce a concrete with 23 MPa target strength would be 1:1.95:3.49.

## Method validation

Another instance is that if a specified SPC mix-design of 1:2:4 is prescribed to be batched by weight with 12 mm coarse aggregate size. The binder-aggregate ratio would be 0.167, this point will be located on the weight batch 12 mm aggregate curve and the target strength determined on Fig. 3. Another point on same Fig. 3 would be located on the “Vol Eq 12mm” curve that corresponds to that target strength on the earlier curve and the binder aggregate ratio determined, which is 0.223. The inverse of 0.223 would be 4.484 which would be equivalent to the sum of the fine and coarse aggregate ratios. Since the coarse to fine aggregate ratio would be 1.776, then the mix-design by volumetric batch that would produce a concrete characteristic, as though the original mix-design of 1:2:4 was batched by weight, would be 1:1.62:2.87. Therefore, the volumetric batch's mix ratio/design of SPC mix-design 1:2:4 prescribed to be batched by weight, would be 1:1.62:2.87. This validates the volume equivalent of the weight-batched design-mix of 1:2:4 (1:1.6:2.88) as shown in Table 1.

## Conclusion, reccomendation and limitations

From the results and discussions, the following were the conclusions made:

➢ Batching the same mix-design by weight will produce better quality concrete than when batched by volume.
➢ There exist a possibility of designing a mix to be batched by volume that would be equivalent to any specified standardised prescribed mix-design by weight as long as the design strength is not greater than 30 MPa.
➢ This study would go a long way in addressing the reduced quality of concrete produced in Nigerian structural concrete applications due to the predominance of the volumetric batching methods employed instead of batching such mix desings by weight. This is achieved by still enjoying the convenience of batching by volume with a derived mix-design from the originally specified mix-designed by weight. The weight equivalent of this derived mix-design will be the same to the originally specified mix design. This will translate to better structural intergrity of buildings within the study area.

This study hereby recommend the following:

➢ Figs. 2 and 3 could be used as a chart for deriving a standardised prescribed concrete mix-design by volume that would be equivalent to a specified mix-design by weight as long as the target strength is not beyond 25 MPa.
➢ This same study be repeated on a matrix combination of fine and coarse aggregate that is available within a locality so as to use the strength design charts as Table 1, Figs. 2 and 3 of the selected aggregates to determine the volume equivalent mix-design that would be equivalent to the specified mix-design by weight. Though the difference is expected to be minute with the charts generated in this study.
➢ Further research is recommended to perform this same study utilising other coarse aggregate sizes between 40 mm and 22 mm (specified SPC sizes in [4,5] for other concrete applications.
